# Reducing Agents Aid in the Texturization of Pea Protein Isolate During High Moisture Extrusion

**DOI:** 10.1111/1750-3841.70797

**Published:** 2026-01-13

**Authors:** Joshua B. Bernin, Preston Watanabe, Brennan Smith, Ryan J. Kowalski, Girish M. Ganjyal

**Affiliations:** ^1^ School of Food Science Washington State University Pullman Washington USA; ^2^ USDA‐ARS, Food Processing and Sensory Quality Research New Orleans Louisiana USA; ^3^ Hills Pet Nutrition, Inc. Topeka Kansas USA

**Keywords:** extrusion, high moisture meat analogs, protein, texturization

## Abstract

One of the leading hypotheses of plant protein texturization during extrusion is the formation of disulfide bonds during the extrusion process. This study aimed to gain a deeper understanding of the role of disulfide bonds during the texturization of plant protein high moisture meat analogs. Pea protein was blended with three reducing agents, sodium metabisulfite, cysteine, and glutathione, at varying levels of inclusion. The blends were extruded using a co‐rotating twin‐screw extruder at two different temperature settings of 130°C and 150°C. The feed rate (60 g/min), screw speed (100 rpm), and moisture content (60% w.b.) were kept constant. The extrudates were evaluated for polymeric protein size exclusion, disulfide, and thiol bond quantification, integrity index analysis, and anisotropic index. The reducing agents cleaved disulfide bonds and significantly affected the structure, texture, and integrity index of the extrudates. The reducing agents also had varying effects on the extrudate and the flow of the melt, with the best product obtained with 0.05% glutathione inclusion extruded at 150°C. Although the reducing agents had a relatively small impact on the disulfide bonds, they had a major impact on the physical characteristics of the product and the crosslinking of proteins.

## 1 Introduction

The food industry has seen a recent increase in plant‐centric diets, increasing the demand for plant‐based meat analogs (J. Zhang et al. 2021). Many people have begun to change their lifestyles from getting protein from animal sources to plant sources because of environmental, health, and ethical reasons (Rubio et al. 2020; Y. Zhang and Ryu 2023). Most plant‐based meat analogs in the market are made from wheat, soy, and, just recently, pulse proteins (Chen et al. 2011; Richter, Montero, et al. 2024; Richter, Smith, et al. 2024; Richter, Watanabe, et al. 2024; Snel et al. 2023; Vatansever et al. 2020; Wagner and Ganjyal 2024).

High moisture meat analogs (HMMA) are one of the few emerging products that are produced using a twin‐screw extruder (Gu and Kowalski 2017). HMMA is different from the other products produced using the same method because it utilizes a cooling die that allows for the formation of a fibrous, layered product that closely resembles animal meat products (Choton et al. 2020; Ismail et al. 2020; Ryu 2020; Sandoval Murillo et al. 2019). Research has been conducted to understand the mechanisms of texturizing plant proteins through extrusion, although the topic has not been comprehensively explored. Many studies have focused on the importance of non‐covalent interactions and the formation of covalent bonds, particularly the role of disulfide bonds in creating fibers and imparting a meat‐like texture (Beniwal et al. 2021; Chen et al. 2011; Junjie et al. 2021; Osen et al. 2015, 2014; Richter, Smith, et al. 2024; Schmid and Torley 2022; Snel et al. 2023). Some researchers attribute the fibrous meat‐like texture directly to the disulfide bond formation in extrusion (Chiang et al. 2019; Osen et al. 2015). However, there is still debate in the scientific community about how disulfide bonds affect the formation and texture of the HMMA.

If disulfide bonds play a crucial role in forming fibers, the question arises regarding how reducing agents might affect protein texturization during extrusion processing. These reducing agents should decrease the fibrousness of the extrudates due to the cleaving action of the disulfide bonds in the extrudate. Richter, Smith, et al. (2024) evaluated the inclusion of low amounts (0.01%–0.75%) of sodium metabisulfite, glutathione, and cysteine in wheat protein. They reported that under select conditions, there was increased fibrousness with cysteine and sodium metabisulfite at an inclusion level of 0.50%. Richter, Smith, et al. (2024) proposed that reducing agents break the disulfide bonds early in the extruder system, enhancing the melt flow during extrusion and promoting cross‐linking between proteins (Wagner et al. 2024, 2025). Dai and An (2022) and Peng et al. (2022) added cysteine to soy and pea protein, respectively. They found that the addition of reducing agents in small quantities (<0.12%–0.15%) created more fibrous products. Dai and An (2022) concluded that cysteine in larger amounts had a negative impact on the fiber formation and texture of products using pea protein. Other reducing agents have not been researched for their impact on the texture and fiber formation of HMMA using pea protein. Thus, this study aims to understand the effect of sodium metabisulfite, glutathione, and cysteine on pea HMMA products.

## 2 Materials and Methods

### 2.1 Materials

Pea protein (Puris 860) was procured from Puris (Minneapolis, MN, USA). Cysteine and glutathione were purchased from BulkSupplements.com (Henderson, NV, USA), LD Carlson Company (Kent, OH, USA), and sodium metabisulfite was purchased from MP Biomedicals (Solon, OH, USA). The pea protein (82.5% d.b.) was mixed with the reductants at 0.01%, 0.05%, 0.25%, 0.50%, and 0.75% (w/w) inclusion levels and adjusted to 10% (w.b.) moisture using a Hobart paddle mixer (Model A‐200, The Hobart Mfg. Co., OH, USA) for 5 min to ensure even distribution of the reducing agents and water. The batches were then placed into polyethylene bags and refrigerated for <16 h to ensure the equilibrium of moisture in the sample.

### 2.2 Extrusion Processing

Extrusion processing was conducted by the method previously described by Richter, Smith, et al. (2024), using a co‐rotating twin‐screw extruder (Model TSE 20/40, 7.5HP, Brabender GmbH & Co. KG, Duisburg, Germany) with an L/D ratio of 40:1, with a diameter of 20 mm (Richter, Montero, et al. 2024; Wagner et al. 2024). Beginning at the feeding port to the die adapter, the temperature profiles used were 50°C, 50°C, 130°C, 150°C, 150°C and 50°C, 50°C, 130°C, 130°C, 130°C. A modified cooling die (Brabender GmbH & Co. KG, Duisburg, Germany) 7 mm in height, 22 mm in width, and 300 mm in length, was attached to the die adapter. The cooling die was set at 50°C using a water circulator (Julabo 200F, Type 10‐22‐000, Brabender GmbH & Co. KG, Duisburg, Germany) with a propylene glycol and water mixed in a 50:50 ratio as a coolant.

The raw material was fed into the extruder in zone one using a twin‐screw volumetric feeder (DDSR20‐5, C.W. Brabender Technologies, Inc., South Hackensack, NJ, USA). The feed rate was adjusted to 60 g/min. Deionized water was pumped into the extruder in between zones one and two to achieve a final moisture content of 56% (w.b.) using a peristaltic water pump (MasterFlex L/S, Model No. 07528‐10, with an Easy‐Load II Head, Model 77200–60, Cole Palmer Instrument Company, Vernon Hills, IL, USA). Samples were then collected for 5 min after the pressure and torque had stabilized. Samples were first stored in airtight polyethylene bags at 4°C until analyzed. A portion of the samples was then freeze‐dried and milled using a Udy cyclone mill (3010‐030, UDY Corp., Fort Collins, CO, USA) with a 0.05 mm screen. Extrusion processing was conducted in duplicate. System parameters, such as torque and pressure, were continually recorded during processing using an Intelli‐Torque Plasti‐Corder data acquisition system (C.W. Brabender Instruments, Inc., South Hackensack, NJ, USA). For each sample, ten data points were used for data analysis, and they were randomly selected after the extruder had been stabilized. Specific mechanical energy (SME) was analyzed using a method described by Kowalski et al. (2018).

### 2.3 Polymeric Protein Extraction

Polymeric protein extraction was conducted as per Smith et al. (2010). Proteins were extracted or reduced into soluble (SP), insoluble (IP), and residue proteins (RPs). The SP fraction was extracted by adding 1 mL of 50 mM sodium phosphate buffer, pH 7, containing 1% (w/v) sodium dodecyl sulfate (SDS) to 10 mg of the dried and milled extrudate. This sample was vortexed for 15 min and centrifuged at 15,000 × *g* for 5 min. The supernatant was collected, and the pellet was then subjected to another extraction step. After the second extraction step, both supernatants were combined and filtered using an assembled filter unit with a 0.4 µm syringeless spin filter and spun at 5000 × *g* for 5 min, resulting in the SP fraction. For the IP fraction, 1 mL of the previously described buffer was added to the remaining pellet. The sample was then vortexed for 15 min and then ultrasonicated using a 50 W 20 kHz ultrasound generator (FisherbrandTM Model 50 Sonic Dismembrator, Thermo Fisher Scientific, Waltham, MA, USA) at 40% amplitude for 30 s, then placed on ice for 30 s. The sample was centrifuged for 5 min at 15,000 × *g*, and the supernatant was collected. The pellet was then subjected to the extraction process, and the supernatant was pooled with the first duplicate. The combined supernatant was then filtered as described above to obtain the IP fraction. For the RP fraction, 1 mL of 50 mM sodium phosphate buffer, pH 7, containing 1% SDS (w/v) and 2% β‐mercaptoethanol (w/v) was added to the remaining pellet. This sample was then subjected to the same treatment as the SP and IP fractions (Smith et al. 2010). There was no pellet remaining after the RP extraction.

Extractants were analyzed by size exclusion chromatography (SEC) on an Agilent 1260 HPLC with a binary pump and a 300 × 7.8 mm BioSep‐SEC‐s4000 column with a particle size of 5 µm (Phenomenex, Torrance, CA, USA). Sodium phosphate buffer (50 mM, pH 7) containing 1% (w/v) SDS was used as the mobile phase with a 1 mL/min flow rate. The column temperature was 40°C with an injection volume of 10 µL. Absorbance was then measured at 210 nm using a diode array detector (DAD). Data was collected for 20 min. Graphs were plotted using OriginPro (Version 2023b, Origin Lab Corporation, Northampton, MA, USA).

### 2.4 Sulfhydryl Groups and Disulfide Bonds

The sulfhydryl groups and disulfide bonds were quantified as per Richter, Smith, et al. (2024). Samples were spectrophotometrically analyzed using Ellman's reagent. The samples were shaken in 10 mL Tris‐Gly buffer (0.086 M Tris, 0.09 M glycine, 0.04 M EDTA, pH 8.0) and 8 M urea for 16 h at room temperature. The sulfhydryl content was analyzed by adding 4 mL Tris‐Gly buffer and 0.05 mL Ellman's reagent to 1 mL of the protein solution and measuring the absorbance at 412 nm after 5 min. The total SH content was analyzed by adding 4 mL Tris‐Gly buffer and 0.05 mL β‐mercaptoethanol to 1 mL of protein solution, and allowed to sit for an hour at room temperature. Then, 10 mL 12% trichloroacetic acid was added to the solution, sat for 1 h at room temperature, and centrifuged at 3230 × *g* for 10 min. To remove the β‐mercaptoethanol, the precipitate was suspended twice in 12% trichloroacetic acid and centrifuged at 3230 × *g* for 10 min. The pellet was then dissolved in 10 mL Tris‐Gly buffer and 0.04 mL Ellman's reagent. The absorbance was then measured at 412 nm after 5 min.

Sulfhydryl group quantification: µM SH/g  =  73.53*A*D/C

Disulfide group quantification: µM SS/g  =  (total SH − SH)/2

With A being the absorbance at 412 nm, C being the sample concentration (mg/ml), and D representing the dilution factor.

### 2.5 Hardness and Anisotropic Index

The hardness and anisotropic index (AI) of the HMMA extrudates were evaluated using a method described by Richter, Smith, et al. (2024). TA‐XT plus C texture analyzer (Stable Micro Systems Ltd, Godalming, United Kingdom). For each analysis, the samples were cut into square pieces with a width of 22.5 mm. Hardness was quantified by a twofold compression to 50% of the sample height using a cylindrical probe with a diameter of 5 cm and a speed of 1 mm/s. The AI was calculated as the ratio between the longitudinal and transversal cutting forces (Beniwal et al. 2021). Longitudinal and transversal hardness were quantified by cutting the sample with a guillotine blade (0.4 mm thick) parallel and perpendicular to the flow of the extrudate. The speed of the blade was set to 1 mm/s. Ten replicates were measured per sample for both tests.

### 2.6 Integrity Index

The integrity index was conducted per Wagner et al. (2024). Briefly, 5 g of sample (dry basis) was placed in a flask with 30 mL of distilled water and placed in a water bath at 80°C for 30 min. The samples were then autoclaved at 121°C for 15 min. The samples were then placed in 100 mL of distilled water and mixed using an overhead stand mixer at 2000 rpm for 1 min (Unilab OS20‐B, Geneva, Switzerland). The sample was then passed through a 20‐mesh sieve and rinsed with distilled water for 1 minute. The sample was then dried at 105°C for 24 h (Y. Zhang and Ryu 2023). The following equation was used to calculate the integrity index.

$$\mathrm{Integrity}\;\mathrm{index}=\frac{\mathrm{Dry}\;\mathrm{residue}\;\mathrm{wt}.}{\mathrm{Sample}\;\mathrm{wt}.}$$

## 3 Results and Discussion

### 3.1 Visual Assessment

**FIGURE 1:**
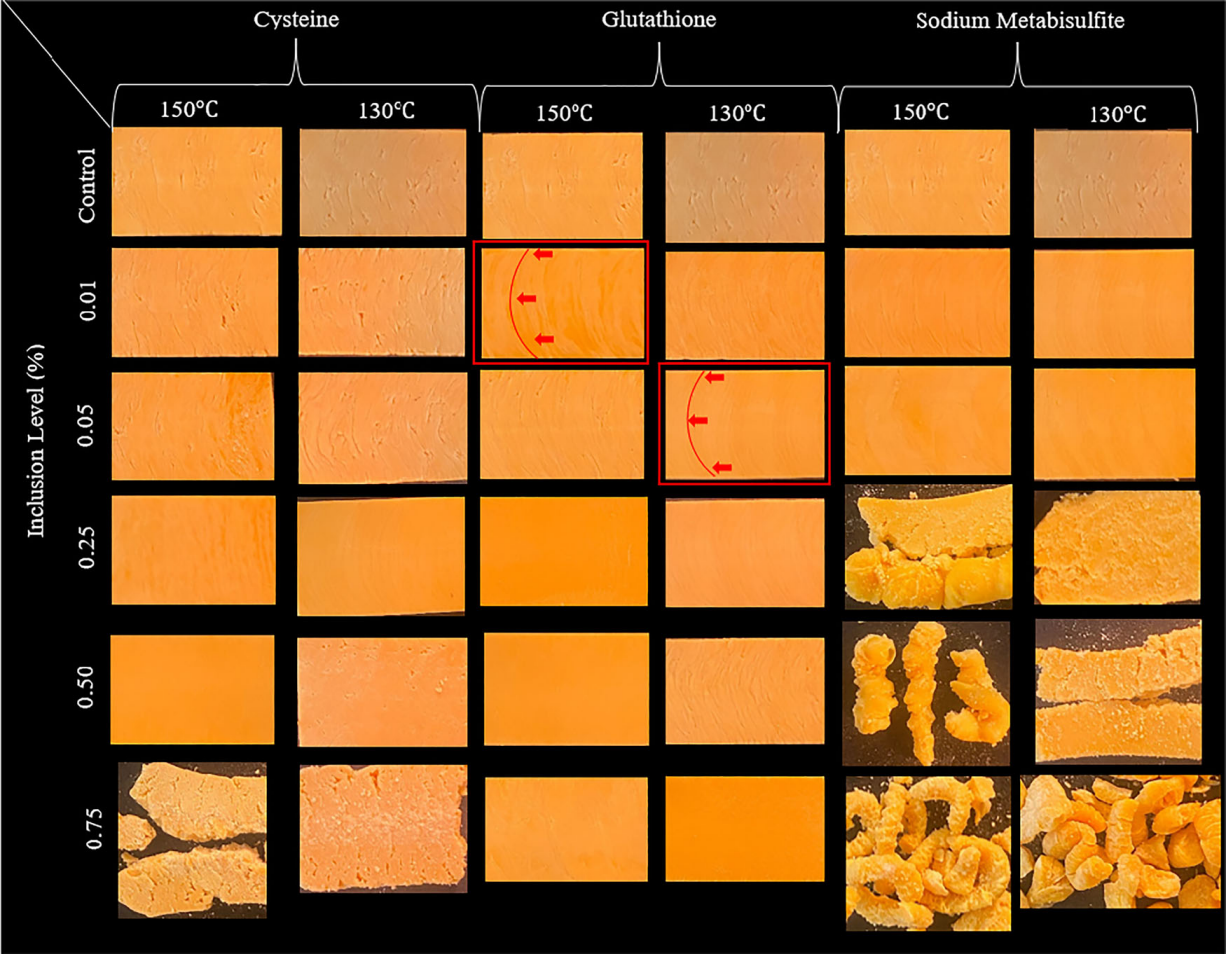
Photographs of pea protein high moisture mean analogs (HMMA) with the inclusion of cysteine, glutathione, and sodium metabisulfite at various concentrations and temperatures. The highlighted samples show a defined laminar flow. In the “Control” row, all three photographs for 150°C are of the same sample, and all three photographs for 135°C are of the same sample. The control photographs are presented in this way for ease of comparison.

Photographs of pea HMMA containing cysteine, glutathione, and sodium metabisulfite are shown in Figure 1. The reductant, temperature, and inclusion levels all impacted the quality of the product. The reductants are hypothesized to have lowered the phase transition temperature, resulting in a more homogeneous product with more tightly packed fibers than the control. This decrease in the phase transition temperature consequently improved the flow behavior of the melt through the extrusion system and the cooling die. This is evident in the products containing lower inclusion levels of reductants, similar to those extruded at a higher temperature with smaller inclusion levels. When looking strictly at the reducing agents used, sodium metabisulfite had the most significant impact on the quality and potentially the phase transition temperature compared to cysteine and glutathione. This is due to the metabisulfite ion being hydrolyzed when water is added to bisulfite, which then interacts and reduces disulfide bonds in the protein (Richter, Smith, et al. 2024). This would happen sooner in the extrusion process, as cysteine and glutathione need more energy to reduce the disulfide bonds in the protein (Dai and An 2022). This results in a change in the melt behavior in the die and creates a potentially different heat transfer rate than glutathione and cysteine. Once the hotter material leaves the cooling die, the water evaporates, leading to a slightly expanded and irregular product.

Sodium metabisulfite had the most effect on the texture and extrudability of the protein, followed by cysteine and glutathione. Glutathione had the least impact on the product but still showed improved texture and flow. As shown in Figure 1, glutathione produced a more homogeneous and cohesive product with numerous tight fibers, which could be extruded at all inclusion levels. Cysteine had a similar effect as glutathione until an inclusion level of 0.75%, where the product was dry and non‐cohesive. The sodium metabisulfite also showed a cohesive and fibrous structure up to the inclusion level of 0.25%. This suggests that the cooling in the die was insufficient and created a channel of semi‐molten material in the middle of the solidified product. As seen in inclusion levels over 0.50% for cysteine and over 0.05% for sodium metabisulfite, the water evaporated once the hot material left the die, leading to a shapeless, expanded, and dry product. These results agree with Richter, Smith, et al. (2024), who used wheat protein mixed with sodium metabisulfite, glutathione, and cysteine. They found that the flow improved with the addition of reductants and that sodium metabisulfite had the most significant effect on the visual appearance, fibrousness, and protein complexation, followed by cysteine and then glutathione. This is due to the different reduction mechanisms compared to glutathione and cysteine. The products with smaller inclusion levels at higher temperatures had similar texturization to the same reductant at lower temperatures and higher inclusion levels (Richter, Smith, et al. 2024). The inverse relationship between the reducing agents and the fibrousness is due to too many disulfide bonds being broken, and therefore, the fibers cannot form as easily (Dai and An 2022). Dai and An (2022) added cysteine to pea protein and found that adding cysteine in concentrations up to 0.15% to pea protein increased the fibrousness. They hypothesized that the reason for this effect is that cysteine promotes the formation of new disulfide bonds and non‐covalent bonds by promoting cross‐linking between proteins. This same observation can be seen in the present study; however, it can be observed that 0.25% of cysteine can be added before it creates a non‐homogeneous product.

Samples containing sodium metabisulfite behaved differently from those containing glutathione and cysteine. This is because sodium metabisulfite acts as a reducing agent that modifies cysteine residues and disrupts disulfide bonds, rather than directly generating free thiol (S‐H) groups. Richter, Smith, et al. (2024) reported that there was a significant increase in SH groups pre‐extrusion in wheat protein. This is evidence of sodium metabisulfite actively reducing disulfide bonds before extrusion. This mechanism could also be occurring in this study, as the prepared blends were stored in a refrigerator for over 16 h. This would provide time for the sodium metabisulfite to begin to reduce disulfide bonds in the protein. This would significantly impact the pea protein because of the reduced concentrations of disulfide‐forming amino acids, as there won't be enough disulfide bonds to support the fiber formation.

### 3.2 Extrusion Response Variables

Extrusion response variables, including pressure, torque, and SME are displayed in Table 1. All extrusion response variables were significantly (*p* < 0.05) affected by temperature, the inclusion level of the reductant, and the interaction between the reductant and the temperature. The torque ranged from 2.44 (0.05% cysteine extruded at 150°C) to 8.01 Nm (0.25% sodium metabisulfite extruded at 130°C), the pressure ranged from 89.12 psi (0.05% cysteine extruded at 150°C) to 532.35 psi (0.25% sodium metabisulfite extruded at 150°C), the SME ranged from 54.61 kJ/kg (0.05% cysteine extruded at 150°C) to 179.13 kJ/kg (0.25% sodium metabisulfite extruded at 130°C).

**TABLE 1:** The system response variables, including pressure, torque, and specific mechanical energy (SME), are measured during highmoisture extrusion containing cysteine (C), sodium metabisulfite (S), and glutathione (G). Data is represented as mean ± standard deviation, with letters denoting significant differences (*p* < 0.05) (*n* = 20).

Temperature (°C)	Inclusion level (%)	Pressure (PSI)	Torque (Nm)	SME (kJ/kg)
150	Control	124.77 ± 9.53^i–k^	3.38 ± 0.61^i–l^	075.63 ± 13.53^h–k^
	C.01	089.12 ± 5.80^k^	2.44 ± 0.05^n^	054.61 ± 1.23^k^
	C.05	108.84 ± 18.08^i–k^	3.26 ± 0.66^l–m^	072.87 ± 14.48^i–k^
	C.25	155.00 ± 47.03^g–j^	4.79 ± 0.27^d–e^	107.10 ± 6.05^d–f^
	C.50	221.37 ± 15.78^e–f^	4.08 ± 1.59^e–k^	110.47 ± 35.53^e–i^
	C.75	243.26 ± 12.99^d–e^	5.06 ± 0.11^d^	113.06 ± 2.56^c–e^
	G.01	202.23 ± 29.27^e–h^	4.43 ± 0.39^d–g^	099.06 ± 8.64^e–g^
	G.05	137.37 ± 13.06^h–k^	3.89 ± 0.66^f–l^	086.92 ± 14.74^f–j^
	G.25	141.37 ± 29.75^g–k^	3.41 ± 0.12^j–l^	076.22 ± 2.70^h–k^
	G.50	112.60 ± 38.59^i–k^	3.41 ± 0.25^i–l^	076.25 ± 5.64^h–k^
	G.75	115.86 ± 41.12^i–k^	3.29 ± 0.70^l^	073.45 ± 15.62^i–k^
	S.01	374.98 ± 23.94^b^	5.87 ± 1.78^b^	131.34 ± 39.81^b–d^
	S.05	119.35 ± 5.43^i–k^	6.33 ± 2.58^a^	141.54 ± 57.78^b^
	S.25	532.35 ± 79.60^a^	7.93 ± 2.76^b^	177.37 ± 61.60^a^
	S.50	350.30 ± 30.09^b–c^	5.82 ± 1.87^e–i^	163.23 ± 41.90^b–d^
	S.75	332.84 ± 43.08^b–c^	4.18 ± 0.54^d–f^	093.55 ± 12.02^e–i^
130	Control	146.74 ± 14.67^g–k^	3.62 ± 0.65^g–l^	081.03 ± 14.55^g–j^
	C.01	104.21 ± 8.60^j–k^	2.49 ± 0.13^m–n^	055.62 ± 2.86^k^
	C.05	132.12 ± 19.70^i–k^	3.33 ± 0.60^k–l^	074.46 ± 13.38^h–k^
	C.25	139.81 ± 25.31^g–k^	4.48 ± 0.26^d–g^	100.12 ± 5.84^e–g^
	C.50	213.56 ± 16.09^b^	4.22 ± 1.66^d–h^	090.94 ± 3.02^e–h^
	C.75	340.47 ± 69.66^b–c^	4.23 ± 0.36^e–j^	094.53 ± 8.11^e–i^
	G.01	204.72 ± 10.14^e–g^	4.17 ± 0.18^e–k^	093.21 ± 3.95^e–i^
	G.05	140.60 ± 16.23^g–k^	3.76 ± 0.39^d–h^	084.10 ± 8.65^f–j^
	G.25	172.30 ± 12.78^f–i^	3.84 ± 0.41^f–l^	085.92 ± 9.15^f–j^
	G.50	109.50 ± 15.09^i–k^	3.39 ± 0.19^h–l^	075.72 ± 4.23^h–k^
	G.75	101.49 ± 53.77^j–k^	2.94 ± 0.34^h–l^	065.82 ± 7.62^j–k^
	S.01	351.05 ± 22.48^b–c^	6.01 ± 1.45^d–h^	134.41 ± 32.52^b–c^
	S.05	159.02 ± 3.96^f–j^	6.42 ± 2.34^c^	143.61 ± 5.22^b^
	S.25	494.19 ± 13.34^a^	8.01 ± 3.05^b^	179.13 ± 6.82^a^
	S.50	353.12 ± 50.14^b–c^	5.77 ± 1.83^e–l^	129.10 ± 4.09^f–i^
	S.75	289.51 ± 77.22^c–d^	3.96 ± 0.61^f–l^	088.57 ± 13.68^f–j^

There were identifiable trends between the SME and the inclusion levels of reducing agents as they increased. When cysteine was added at levels ranging from 0.01%–0.05%, the torque increased and then leveled off. This interaction could also be observed at both 130°C and 150°C extrusion temperatures, with a slight decrease in torque after 0.25% at the lower extrusion temperature. This is unexpected as the reducing agent was hypothesized to decrease the torque because of the reduction of disulfide bonds between protein molecules and the consequential decrease in protein polymer size (Ek et al. 2020; Kowalski et al. 2018). This change in torque can be attributed to the protein melt becoming dry and increasing the torque, as evident in Figure 1. This is an indication that the hydration properties of the protein change with the increase of reducing agents. The inclusion of glutathione and sodium metabisulfite behaved differently from that of cysteine. In general, when glutathione was added, the torque decreased with the increase of glutathione at both temperatures, which is more aligned with what was expected when increasing amounts of the reducing agent were added. The addition of sodium metabisulfite significantly increased the torque from 5.87 (0.01% extruded at 150°C) to 7.93 Nm (0.25% extruded at 150°C) and then decreased the torque to 4.18 Nm (0.75% extruded at 150°C). This same pattern can be seen with the products extruded at 130°C with slightly higher torque values. In general, these slightly higher torque readings could be observed at lower temperatures, as the lower temperature leads to an increase in the viscosity of the melt.

It is important to note that because the three different reductants behaved differently during the extrusion process, it is difficult to explain their influence on extrusion response variables. Each reducing agent likely has a different action and response to the extrusion process, even when they all cleave the disulfide bonds of proteins. When these bonds are broken, the protein undergoes conformational changes. This could impact the non‐covalent bonds and hydration properties of the protein. These changes then affect the extruder response variables, which are observed as higher or lower torque and pressure readings. As discussed previously, cysteine may help the protein bind more water, thereby increasing the viscosity of the melt. Conversely, glutathione lowered the viscosity of the melt, therefore improving the flow. Because adding cysteine appeared to enhance the texture of the product without decreasing the torque, it can be inferred that factors other than melt viscosity contribute to the observed changes. Conversely, some reducing agents may influence the melt, decreasing viscosity because they reduce the disulfide bonds between proteins, resulting in lower average molecular weights.

### 3.3 Polymeric Protein Size Extraction

**FIGURE 2:**
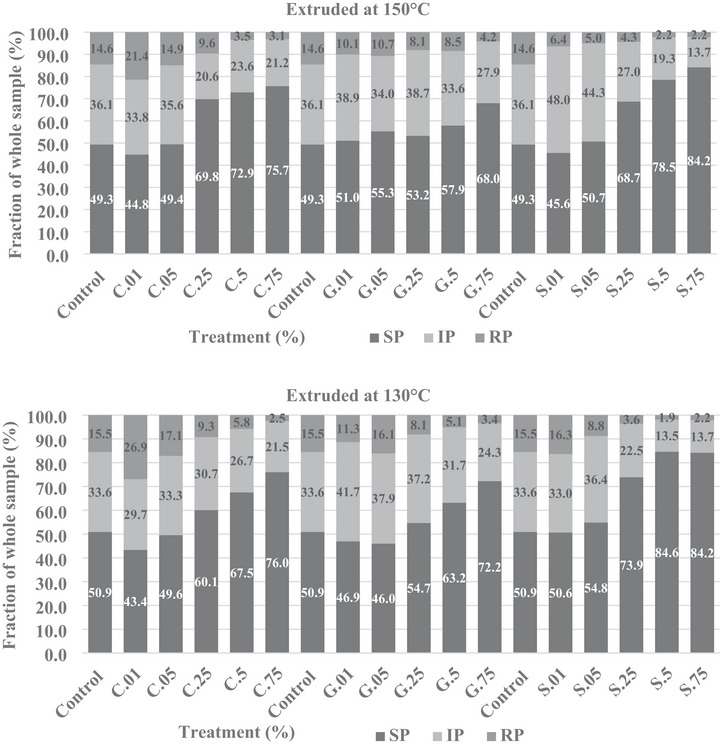
The distribution of soluble proteins (SP), insoluble proteins (IP), and residue proteins (RP) in extruded pea protein containing cysteine (C), glutathione (G), and sodium metabisulfite (S) using an HPLC.

Polymeric protein extraction of pea protein concentrate with cysteine, glutathione, and sodium metabisulfite is shown in Figure 2. The lower molecular weight protein fraction was contained in the soluble protein fraction (SP). In contrast, larger molecular weight protein polymers and entangled proteins were found in the insoluble protein (IP) fraction, and the largest disulfide‐linked polymeric protein fraction was found in the RP fraction.

For all reducing agents used, the relative quantity of the RP fraction was lowered with the increased concentration of the reducing agents. Glutathione had the least effect on the protein while reducing the RP fraction and increasing the SP fraction. The IP fraction remained relatively constant, except at the largest inclusion level of 0.75%, where it decreased. The cysteine moderately affected the pea protein extrudates, decreasing the RP and IP fractions while the SP fraction increased. The sodium metabisulfite had the greatest impact on the pea extrudates, decreasing the RP and IP fractions the most at all levels (Table S1). This could be due to the unique method of reduction compared to cysteine and glutathione, which utilize a free thiol group to reduce disulfide bonds. Sodium metabisulfite and cysteine seem to have created a drier product at the higher inclusion levels (0.50% and 0.75% inclusion). The barrel temperature of the extruder had no noticeable effect on the fraction of protein. Richter, Smith, et al. (2024), using the same reducing agents, found that the reducing agents increased the RP fractions of the protein and concluded that the reducing agents helped denature the proteins earlier in the extrusion process and helped create larger polymeric proteins. They also hypothesized that the reducing agents facilitated the flow through the extruder and into the die and that this mechanism was responsible for the increased texturization of the extrudates. The improvements to the extrudate observed in this study may also be attributed to wheat having a high degree of cross‐linking, making reducing agents necessary to achieve a better flow profile. Their results do not corroborate the results in this study, as the higher the inclusion level of the reducing agent, the less structured the product became. The lack of structure and the apparent change in hydration properties of the pea extrudates in this study could be a result of conformational changes in the pea protein. These changes then allow the pea protein to hold more water, creating extruding conditions that are not optimal for all concentrations of reducing agents. The extrusion temperatures used in this study are also considered to be lower. These lower temperatures could also slow the chemical interactions, affecting the reduction of disulfide bonds and the alignment of the protein in the barrel.

### 3.4 Sulfhydryl Groups and Disulfide Bonds

**FIGURE 3:**
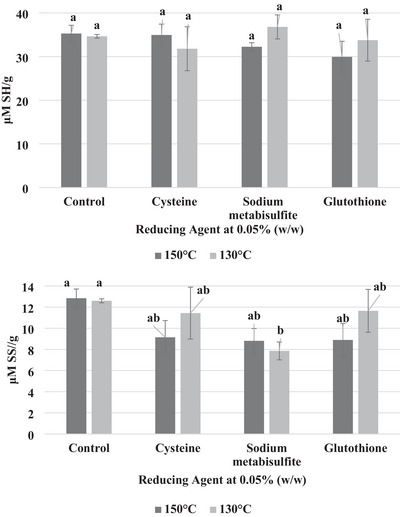
Disulfide bonds and thiol group contents in extruded pea protein at 0.05% (w/w) reducing agents. Error bars represent standard deviations (*n* = 3), and the different letters represent significance (*p* < 0.05), µM SH/g. There were no significant differences (*p* < 0.05) between samples when measuring the sulfhydryl groups (µM SH/g).

The sulfhydryl groups and disulfide bonds are shown in Figure 3. The reducing agents did not increase the ─SH groups at any level, and the increase was not statistically significant (*p* < 0.05). There were minor differences between the reducing agents, specifically between glutathione and the other two reducing agents. There were significant differences between the reducing agents and the disulfide bonds. The reducing agents decreased the disulfide bonds, with sodium metabisulfite having the most significant impact, as seen in Figure 3. Cysteine and glutathione had similar effects on the HMMA. Combining polymeric protein size exclusion results and disulfide and SH bond testing, it is clear that the reducing agents reduced the disulfide bonds present in the protein matrix. However, we do not know if the disulfide bonds that were reformed during extrusion are interprotein or crosslinked with another protein. Similar thiol group quantification among all the reducing agents and the polymeric protein size exclusion data showed a decrease in larger polymeric protein components. This means that the sulfur‐containing amino acids are creating thiol groups, or the disulfide bonds that are created are interprotein and do not form crosslinking disulfide bonds with other protein molecules. It is important to note that another reason the thiol bond quantification remains the same for samples containing glutathione and cysteine is that the thiol groups in these reducing agents are being analyzed along with the thiol groups of the protein. However, because of the similar thiol bonds to the control, it is unlikely that this is the case.

### 3.5 Hardness and AI

The hardness and degree of texturization data are provided in Table 2. There were no obvious patterns for hardness that could be observed throughout the dataset. However, it can be seen that the products extruded at 150°C with cysteine and sodium metabisulfite exhibited increased hardness with an increase in the reducing agent inclusion level. The sodium metabisulfite with inclusion levels over 0.05% did not form a cohesive product (Figure 1) but instead created a heterogeneous product. This results in an inability to quantify the product hardness. This same situation occurred with cystine at concentrations above 0.50% inclusion. The glutathione did not show any specific pattern. The hardness plateaued between 0.01% and 0.25%, and the hardness decreased between 0.50% and 0.75% (Table 2). A similar trend was reported by Peng et al. (2022), who included cysteine in various concentrations added to pea concentrate and observed that there were no obvious continuous trends found in the data. However, they found that there was an increase in hardness in samples containing lower amounts of cysteine, followed by an eventual decrease with the increased inclusion of cysteine (Peng et al. 2022). Dai and An (2022) added cysteine to soy and wheat gluten and observed similar trends for hardness. In general, for extrudates with cysteine inclusion, the hardness increased at 130°C, while sodium metabisulfite inclusion decreased the hardness at an inclusion level of 0.01% and 0.05% at the lower temperature.

**TABLE 2:** The hardness and degree of texturization of high moisture meat analogs containing cysteine (C), sodium metabisulfite (S), and glutathione (G). Data is represented as mean ± standard deviation, with letters denoting significant differences (*p* < 0.05) (*n* = 20).

Temperature (°C)	Inclusion level (%)	Hardness (N)	Anisotropic index (AI)
150	Control	319.14 ± 43.98^h–i^	0.83 ± 0.07^a–d^
	C.01	322.87 ± 30.14^g–i^	0.69 ± 0.05^d^
	C.05	326.55 ± 14.23^g–i^	0.77 ± 0.04^b–d^
	C.25	348.35 ± 32.18^d–h^	0.85 ± 0.15^a–d^
	C.5	326.67 ± 31.91^g–i^	0.95 ± 0.09^a^
	C.75	n/a	n/a
	G.01	368.36 ± 22.32^a–g^	0.79 ± 0.12^b–d^
	G.05	326.28 ± 20.43^g–i^	0.76 ± 0.07^b–d^
	G.25	402.60 ± 61.69^a^	0.81 ± 0.07^a–d^
	G.5	354.50 ± 29.58^b–h^	0.85 ± 0.04^a–d^
	G.75	304.57 ± 29.71^i^	0.91 ± 0.06^a–b^
	S.01	354.77 ± 24.99^b–h^	0.81 ± 0.04^a–d^
	S.05	388.95 ± 22.85^a–d^	0.89 ± 0.14^a–c^
	S.25	n/a	n/a
	S.5	n/a	n/a
	S.75	n/a	n/a
130	Control	385.66 ± 27.93^a–e^	0.74 ± 0.07^c–d^
	C.01	336.87 ± 19.81^i–f^	0.72 ± 0.11^c–d^
	C.05	402.31 ± 29.30^a^	0.76 ± 0.07^b–d^
	C.25	395.55 ± 40.21^a–b^	0.81 ± 0.16^a–d^
	C.5	375.68 ± 33.17^a–h^	0.82 ± 0.10^a–d^
	C.75	338.10 ± 20.60^f–i^	0.80 ± 0.09^a–d^
	G.01	397.31 ± 19.17^a–c^	0.79 ± 0.12^b–d^
	G.05	380.20 ± 49.40^a–f^	0.75 ± 0.07^c–d^
	G.25	391.91 ± 33.68^a–d^	0.82 ± 0.05^a–d^
	G.5	387.65 ± 26.57^a–e^	0.80 ± 0.04^a–d^
	G.75	362.97 ± 26.57^a–h^	0.88 ± 0.08^a–c^
	S.01	354.12 ± 23.29^c–h^	0.79 ± 0.12^b–d^
	S.05	341.81 ± 23.02^e–i^	0.84 ± 0.12^a–d^
	S.25	n/a	n/a
	S.5	n/a	n/a
	S.75	n/a	n/a

In general, when extruded at 130°C, the products with glutathione were harder than the products extruded at 150°C. This increase in hardness at lower temperatures is likely the result of more crosslinked proteins and a more cohesive protein network. The products that could not be evaluated for hardness could be attributed to the insufficient cooling in the cooling die. This insufficient cooling results in water evaporation and, consequently, product expansion, as is evident when sodium metabisulfite is included above 0.05% in Figure 1.

The AI has been used to analyze the direction of fiber formation and assess the fibrousness of the product (Richter, Watanabe, et al. 2024; Wagner and Ganjyal 2024). Peng et al. (2022) reported that products with an AI > 1 are desired, while Beniwal et al. (2021) stated that an AI > 1.5 is considered fibrous, while a product with an AI = 1 is considered homogenous. In our study, no products had an AI above 1, with the largest AI value of 0.95 ± 0.09 showing fiber formation perpendicular to the direction of the flow (Figure 1). The highest AI values were recorded with a cysteine inclusion level of 0.50% and a glutathione inclusion level of 0.75; both were extruded at 150°C. They were not visually evaluated as being the most fibrous products. Therefore, AI might give a good indication of the sample direction of fibers formed. However, it should be known that when interpreting AI, it is important to consider the visual assessment of the product when evaluating fibrousness.

### 3.6 Integrity Index

The integrity index measures the ability of the product to maintain its integrity while being exposed to cooking conditions (Wagner and Ganjyal 2024). The integrity index data is shown in Table 3. The extrudates with cysteine inclusion exhibited an increase in integrity index up to a 0.25% inclusion level, followed by a decrease in integrity index at both extrusion temperatures. The inclusion of glutathione did not change the integrity index of the product. The inclusion of sodium metabisulfite had the most impact on the integrity index. The integrity index decreased and then increased with an inclusion level of 0.75% sodium metabisulfite. This indicates that cysteine enhances the stability of the fibers through the formation of disulfide bonds until it reaches a point where the reducing agent hinders the formation of these bonds. It can also be seen that the samples with the highest integrity index tended to be the most homogeneous (Figure 1). The samples that did not cool adequately in the cooling die, specifically samples extruded with sodium metabisulfite inclusion levels above 0.05%, had a lower integrity index than those that cooled properly. The temperatures tested also did not seem to impact the integrity index of the product.

**TABLE 3:** The integrity index of high moisture meat analogs containing cysteine (C), sodium metabisulfite (S), and glutathione (G). Data is represented as mean ± standard deviation, with letters denoting significant differences (*p* < 0.05) (*n* = 3).

Temperature (°C)	Inclusion level (%)	Integrity index
150	Control	0.3 ± 0.01^a‐b^
	C.01	0.27 ± 0.01^b‐c^
	C.05	0.29 ± 0.01^a‐b^
	C.25	0.3 ± 0.01^a‐b^
	C.5	0.27 ± 0.01^b‐c^
	C.75	0.26 ± 0.03^a‐b^
	G.01	0.3 ± 0.01^a‐b^
	G.05	0.28 ± 0.02^a‐b^
	G.25	0.29 ± 0.01^a‐b^
	G.5	0.28 ± 0.03^a‐b^
	G.75	0.27 ± 0.01^b‐c^
	S.01	0.27 ± 0.04^a‐b^
	S.05	0.27 ± 0.04^a‐b^
	S.25	0.21 ± 0.04^d^
	S.5	0.2 ± 0.03^d^
	S.75	0.25 ± 0.03^b‐c^
130	Control	0.3 ± 0.02^a‐b^
	C.01	0.28 ± 0.01^b‐c^
	C.05	0.28 ± 0.01^a‐b^
	C.25	0.3 ± 0.01^a‐b^
	C.5	0.28 ± 0.01^a‐b^
	C.75	0.26 ± 0.03^b^
	G.01	0.3 ± 0.01^a‐b^
	G.05	0.3 ± 0.02^a‐b^
	G.25	0.29 ± 0.01^a‐b^
	G.5	0.29 ± 0.03^a‐b^
	G.75	0.29 ± 0.01^a‐b^
	S.01	0.3 ± 0.04^a‐b^
	S.05	0.3 ± 0.04^a^
	S.25	0.25 ± 0.04^b‐c^
	S.5	0.17 ± 0.03^c‐d^
	S.75	0.25 ± 0.02^b‐c^

## 4 Conclusion

The reducing agents cysteine, glutathione, and sodium metabisulfite significantly impacted the HMMA extrudates made from pea protein concentrate. Cysteine and Sodium metabisulfite had the most significant impact on the HMMA. All of the reducing agents resulted in smaller protein residues. The reducing agents did help with the texturization of the HMMA products, creating better fibrous structures and a larger AI. However, the integrity index generally decreased with the inclusion of the reducing agents, which is expected as the protein complexation was smaller, resulting in less stable protein chains. However, additional research needs to be conducted to fully understand how the reducing agents affect the flow characteristics of the melt in the cooling die and in more extreme extrusion conditions, such as higher temperatures (< 160°C) and moisture contents ranging from 50% to 70%. In addition, further research is needed to investigate the use of oxidation agents in extrusion, aiming to better understand the role of disulfide bonds in the texturization of plant proteins.

## Author Contributions

**Joshua B. Bernin**: conceptualization, methodology, formal analysis, investigation, data curation, writing – original draft, visualization. **Preston Watanabe**: data curation, writing – review and editing. **Brennan Smith**: investigation, methodology, data curation, visualization, writing – review and editing. **Ryan J. Kowalski**: writing – review and editing, investigation. **Girish M. Ganjyal**: conceptualization, funding acquisition, writing – review and editing, project administration, resources, visualization, supervision.

## Conflicts of Interest

The authors declare no conflicts of interest.

## Supporting information




**Supplementary Material**: jfds70797‐sup‐0001‐TableS1.docx
