# Supplementary material for: Reducing Agents Aid in the Texturization of Pea Protein Isolate During High Moisture Extrusion
Source: J Food Sci. 2026 Jan 13;91(1):e70797. doi: 10.1111/1750-3841.70797 (PMC12797012; doi:10.1111/1750-3841.70797)
Supplement: Supplementary file 1 — Supplementary Material: jfds70797‐sup‐0001‐TableS1.docx [file JFDS-91-0-s001.docx]

**Table 1: ANOVA analysis of polymeric protein size exclusion data.**

| **Variables** | **Source of Interaction** | **MS** | **F** | **p** |
| --- | --- | --- | --- | --- |
| **Soluble Protein** | Cooling Die | 0.061 | 0.001 | 0.980 |
|  | Reducing Agent | 363.410 | 0.955 | 0.472 |
|  | Cooling die*Reducing Agent | 328.390 | 82.099 | 0.518 |
| **Insoluble Protein** | Cooling Die | 21.840 | 0.133 | 0.723 |
|  | Reducing Agent | 741.060 | 185.270 | 0.398 |
|  | Cooling die*Reducing Agent | 425.290 | 106.323 | 0.642 |
| **Residue Protein** | Cooling Die | 19.600 | 1.027 | 0.335 |
|  | Reducing Agent | 209.990 | 52.490 | 0.089 |
|  | Cooling Die*Reducing Agent | 68.843 | 17.210 | 0.498 |
